# The Effect of Curcumin on some of Traditional and Non-traditional Cardiovascular Risk Factors: A Pilot Randomized, Double-blind, Placebo-controlled Trial

**Published:** 2015

**Authors:** Parastoo Mirzabeigi, Amir Hooshang Mohammadpour, Mojtaba Salarifar, Kheirollah Gholami, Mojtaba Mojtahedzadeh, Mohammad Reza Javadi

**Affiliations:** a *Department of Clinical Pharmacy, School of Pharmacy, Tehran University of Medical Sciences, Tehran, Iran. *; b *Pharmaceutical Research Center, Department of Pharmacodynamics and Toxicology, School of Pharmacy, Mashhad University of Medical Sciences, Mashhad, Iran.*; c *Tehran Heart Center, School of Medicine, Tehran University of Medical Sciences, Tehran, Iran.*

**Keywords:** Curcumin, Coronary artery disease, Lipid profile, hs-CRP, Blood glucose

## Abstract

Numerous interventional studies in clinical and preclinical setting stated that intake of curcumin may provide protection against cardiovascular disease. The aim of this trial was investigation of curcumin efficiency on some cardiovascular risk factors in patients with coronary artery disease (CAD). A total of 33 patients with CAD who fulfilled inclusion and exclusion criteria were entered the study. Patients were randomly assigned to receive curcumin or placebo, 500 mg capsules, four times daily for 8 weeks. Lipid profile, blood glucose and high sensitive C-reactive protein (hs-CRP) levels were analyzed at baseline and two months after treatment. Serum levels of triglycerides (P=0.01), LDL-cholesterol (P=0.03) and VLDL-cholesterol (P=0.04) significantly decreased in the curcumin group compared to baseline, without significant changes in total cholesterol, HDL-cholesterol, blood glucose and hs-CRP levels. In all mentioned laboratory parameters, significant difference was not detected between curcumin and placebo. Although curcumin improved some of lipid profile components, it did not show appreciable effect on inflammatory markers in patients with CAD. Therefore, more detailed assessment of metabolic effects or anti-inflammatory activities of curcumin need to perform by extensive human study.

## Introduction

Cardiovascular diseases (CVDs) and their complications are considered as a leading cause of death throughout the world. They can also decrease quality of life. Traditional risk factors of cardiovascular diseases include age, gender, hypertension, dyslipidemia, smoking and diabetes mellitus (1). Among these, dyslipidemia is the atheromatous lesion’s foundation, which accompanied with arterial stenosis. Although these risk factors have a strong association with cardiovascular events; they are insufficient to fully elucidate absolute risk. Therefore the probability of interference of other factors in the pathogenesis of atherosclerosis is considerable (2). Numerous studies have pointed to crucial position of inflammation throughout atherosclerosis stages (3, 4). Studies stated serum concentration of CRP (C-reactive protein) has the most consistent relation with coronary artery disease, so that it could be considered as a nontraditional cardiovascular risk factor (2, 5). Recent studies have concentrated on therapeutic modulation of traditional and nontraditional cardiovascular risk factors in high risk patients in order to prevent cardiovascular events.

Curcumin is a polyphenolic compound and active constituent of the rhizomes of Curcuma longa plant from Zingiberaceae family (6). Due to curcumin’s extensive pharmacological facets including anti-inflammatory, antioxidant, antimicrobial and cancer-preventive properties, it is efficacious in many diseases, including neoplastic, neurological, cardiovascular, metabolic disorder (7-9). Studies also suggest that it has hypolipidemic and hypoglycemic properties. Consequently curcumin might reduce cardiovascular events. Curcumin is known as a safe natural substance with no reported serious adverse effects and doses as high as 12 g/day well tolerated by human subjects (10).

Controversial results have been observed in animal and human studies on lipid profile improvement by curcumin. Few human clinical trials have been conducted in this area, especially in patients with coronary artery disease (CAD). The purpose of this study was to investigate the curcumin efficacy on lipid profile, blood glucose level, and inflammatory markers in patients with CAD. 

## Experimental

*Patients*

The trial performed at the Tehran Heart Center Hospital (Tehran, Iran) between December 2011 and February 2013. Volunteers over 18 years old with a diagnosis of CAD enrolled in the study. CAD diagnosis was confirmed by a cardiologist with performing angiography. Exclusion criteria were patients with 1) active chronic inflammatory or autoimmune disease based on clinical assessments and past medical history; 2) renal (creatinine> 2.0 mg/dl) and, or hepatic failure (aspartate aminotransferase and alanine aminotransferase >2.5 times the upper limit of normal; serum bilirubin > 2.0 mg/dl); 3) infectious disease (as a result of laboratory findings for viral infection or requiring treatment with antibiotics); 4) immunosuppressive, anti-inflammatory and or antibiotic drug use; 5) curcumin sensitivity 6) pregnancy or breastfeeding. Eligible patients signed written informed consent before enrollment. Demographic data, drug history, past medical history, family history, cardiovascular risk factors and laboratory data for each patient collected by a questionnaire. Other initial assessments, including physical examination, vital signs, 12-lead electrocardiogram, measurements of height and body weight, calculation of BMI and smoking status have been done at enrollment. Laboratory evaluations including fasting plasma glucose, lipid profile [total cholesterol (TC), Low-density lipoprotein-cholesterol (LDL-C), High-density lipoprotein-cholesterol (HDL-C), and triglyceride (TG)], liver and kidney function were recorded at baseline and two months after treatment.

*Materials*

Curcumin and curcumin placebo were provided as capsules by Sami Labs Limited Corporation (Bangalore, India). Curcumin C3 complex capsules contain a minimum 95% curcuminoids.

*Study design*

The study protocol was approved by the Ethics Committee of Tehran University of Medical Sciences (TUMS), as a randomized double blind placebo controlled trial. Patients were randomly entered into one of the groups (curcumin or placebo) and were treated with curcumin (1capsule of 500 mg/QID) or placebo in addition to conventional therapies for two months. Initially, patients were provided with 30-day supply of medication (curcumin or placebo). After one month, second supply of capsules was provided. Blood samples from all patients were obtained at the baseline and two months after either curcumin or placebo administration. Blood samples were centrifuged at 4000 rpm for 5 minutes and serum samples separated from fresh blood were stored at -20 °C until analysis time. The serum concentration of hs-C-reactive protein was measured at baseline and two months after treatment by using immunoturbidimetry assays (COBAS Integra 400, Roche Diagnostics). Biochemical tests consisting of blood glucose, TC, LDL-C, HDL-C and TG were measured enzymatically (COBAS Integra 400, Roche Diagnostics) after a 12-hour overnight fast. In addition, kidney and liver function tests were also assessed by enzymatic methods according to the manufacturer’s protocol (COBAS Integra 400, Roche Diagnostics).

*Follow up*

Patients were followed for 2 months after hospital discharge by telephone contact and monthly clinic visits. Investigator evaluated medication compliance by counting the number of capsules returned at study visits. Moreover, safety and tolerability were assessed by patient interview. It should be noted that adverse events including; liver, kidney, gastrointestinal as well as hypersensitivity reaction were recorded specifically during the study.

*Statistical evaluation*

Statistical analyses were performed using SPSS16 (SPSS Inc., Chicago, IL, USA). Statistical data are presented as mean ± Standard Deviation (SD) or as median with Interquartile range (IQR), as appropriate. For determining statistical significance of quantitative variables, comparisons were performed by using t-tests for normal distribution data and Mann-Whitney test for non-parametric data. For paired data, quantitative variables with normal distribution were analyzed by paired-sample t-test and non-parametric data were assessed by Wilcoxon test. For comparing qualitative variables, Chi square test or Fisher's exact test was utilized when appropriate. A p-values < 0.05 were considered statistically significant in all statistical evaluations. 

## Results

*Patient characteristics*

Forty patients with CAD were enrolled in the trial. Among these, Thirty-three patients completed the study, 17 treated with curcumin (n=17) and 16 with placebo (n=16). The two groups were similar in the demographic, clinical, and baseline characteristics (Table 1). Seven patients did not complete the study for reasons such as unplanned revascularization, loss to follow-up or non-compliance.

**Table 1:** Baseline demographic and clinical characteristics of population study

	**Placebo, n=16**	**Curcumin, n=17**	**P**
Age, y, mean ± SD	64.3±8.42	61.5±8.7	0.33^a^
** Male, n (%)**	14 (87.5)	10 (58.8)	0.12^b^
** Weight, Kg**	79.71±1.06	73.30±8.36	0.10^a^
** Height, m**	167.3±7.4	162.61±9.7	0.17^a^
**BMI, Kg/m** ^2^ **, mean ± SD**	28.5±3.67	27.94±3.62	0.20^a^
**Waist circumference, cm**	102.2±6.1	100.6±7.2	0.63^a^
**Active smoker, n (%)**	3(18.8)	6(35.3)	1.00^b^
**Family history of CHD, n (%) **	4(25)	7(41.2)	0.39^c^
Prior history			
** Diabetes, n (%)**	5(31.3)	9(52.9)	0.20^c^
** Hypertension, n (%)**	9(56.3)	13(76.5)	0.47^b^
** Dyslipidemia, n (%)**	8(50)	12(70.6)	0.22^b^
Drug history			
** ACEIs**	4 (25)	7 (41.2)	0.32^c^
** ARBs**	5(31.3)	2(11.8)	0.23^b^
** Statins**	11(68.8)	13(76.5)	0.71^b^
** β-blockers**	9 (56.3)	12 (70.6)	0.39^c^
** Ca-blockers**	3(18.8)	0(0)	0.10^b^
** Nitrate**	10 (62.5)	9 (52.9)	0.58^c^
** Antiplatelet**	14 (87.5)	14 (82.4)	1.00^b^
** Diuretics**	1 (6.3)	3 (17.6)	0.60^b^
** Insulin**	1 (6.3)	4 (23.5)	0.34^b^
** Hypoglycemic agents**	4 (25)	6(35.3)	0.71^b^
Clinical parameters			
** Ef %, mean ± SD**	50.62±6.80	52.18±6.82	0.50^a^
** Heart rate, bpm, mean ± SD**	71.07±9.33	69.47±9.56	0.64^a^
** Systolic BP, mm Hg, mean ± SD**	138.60±16.95	128.82±36.76	0.35^a^
**Diastolic BP, mm Hg, mean ± SD**	80.13±12.42	79.88±13.00	0.95^a^
Baseline blood tests			
** TC, mg** **/** **dl** **, mean ** **±** ** SD **	165.21± 24.36	172.53±45.75	0.60^a^
** HDL-C, mg/dl** **, mean ** **±** ** SD**	43.14±10.82	39.06±10.80	0.24^a^
** LDL-C, mg/dl, mean ± SD**	95.23±20.72	118±44.05	0.10^a^
** VLDL, mg/dl, mean ± SD**	17.85±6.91	28.53±12.38	0.06^a^
** TG, mg/dl, mean ± SD**	108.58±42.27	145.92±52.72	0.06^a^
** FBG, mg/dl, mean ± SD**	105.38±32.27	141.06±55.20	0.05^a^
** Cr, ** **mg** **/** **dl** **, mean ** **±** ** SD**	0.91±0.22	0.89±0.22	0.83^a^
** Urea, mg/dl, mean ± SD**	37.68±11.35	36.79±9.77	0.81^a^
** WBC, /cumm, mean ± SD, ** ** median (IQR)**	8427.50±4505.64, 6900 (5750-10057.50)	8325.29±2051.36, 7900 (7050-9450)	0.23^d^
** PLT-Count, mean ± SD**	190381.8±34487.15	240800±84967.71	0.08^a^
** hs-CRP, mean ± SD, median (IQR)**	0.54±0.58, 0.33 (0.19-0.75)	2.14±5.00, 0.36 (0.14-0.85)	0.79^d^

BMI, body mass index; ACEIs, angiotensin converting enzyme inhibitors; ARBs, angiotensin receptor blockers; Ef, ejection fraction; TC, total cholesterol; HDL-C, high-density lipoprotein; LDL-C, low-density lipoprotein cholesterol; VLDL, very low-density lipoprotein cholesterol; TG, triglycerides; FBG, fasting blood glucose; Cr, creatinine; WBC, white blood cell; CUMM, cubic millimeter; IQR, interquartile range; PLT, platelet.

a t-tests,

b Fisher's exact test,

c Chi square test,

d Mann-Whitney test.

*The effect of curcumin on cardiovascular risk factors*

The results revealed that changes of lipid profile due to the curcumin intervention in comparison with the placebo were not statistically significant (Table 3). As illustrated in Table 2, TC slightly decreased in the curcumin group (19.18%, p=0.30) and the placebo group (8.56%, p=0.17); LDL levels after intervention significantly decreased in the curcumin group (28.87%, p=0.03), and to some extent decreased in the placebo group (14.54%, p=0.15). Comparing baseline with two month samples, VLDL values significantly decreased (p=0.04) in curcumin group, although there was not a significant change in the placebo (p=0.30). HDL levels were trending upward in the curcumin group (5.3%, p=0.20), but the downward trend was observed in placebo group (2%, p=0.77). Serum triglycerides significantly decreased in the curcumin group (2.7%, p=0.01), while non-significantly decreased in the placebo group (2.35%, p=0.61). 

Although FBG decreased in the curcumin group (13.16%, p=0.09) and increased in the placebo group (10.51%, p=0.17), no statistically significant differences were observed between the groups (Tables 2, 3).

As indicated in Table 2, there is no statistically significant difference of serum hs-CRP levels between pre- and post-treatment for patients in the curcumin group (p=0.35). There was no significant between-group difference in hs-CRP measurements (p=0.91). 

**Table2:** The effect of curcumin on cardiovascular risk factors

	Placebo	P	Curcumin	p
	Pre-treatment Post-treatment		Pre-treatment Post-treatment	
TC	165.21± 24.36 151.07±50.31	0.17^a^	172.53±45.75, 154.13±40.10, 172 (141-202) 139 (133-196)	0.30^b^
LDL-C	95.23±20.72 81.38±43.41	0.15^a^	118±44.05, 84.15±28.15, 111 (80-147) 78 (66-99)	0.03^b^
HDL-C	43.14±10.82 42.28±6.54	0.78^a^	39.06±10.80 41.13±9.20	0.20^a^
VLDL	17.85±6.91 17.25±7.31	0.30^a^	28.53±12.38 22.68±8.10	0.04^a^
TG	108.58±42.27 108.75±53.32	0.98^a^	145.92±52.72 120.15±10.47	0.01^a^
FBG	105.38±32.27 116.46±24.96	0.84^a^	141.06±55.20 122.50±35.68	0.09^a^
hs-CRP	0.54±0.58, 0.23±0.18, 0.33 (0.19-0.75) 0.19 (0.11-0.30)	0.20^b^	2.14±5.00, 0.59±0.76, 0.36 (0.14-0.85) 0.22 (0.10-0.83)	0.35^b^

Values are mean ± SD or median (IQR).

TC, total cholesterol; LDL-C, low-density lipoprotein cholesterol; HDL-C, high-density lipoprotein; VLDL, very low-density lipoprotein cholesterol; TG, triglycerides; FBG, fasting blood glucose; hs-CRP, high-sensitive C-reactive protein.

a Paired-sample t-test,

b Wilcoxon test.

*Adverse drug reactions*

During the trial, any side effect was evaluated in study population. Two gastrointestinal (GI) adverse symptoms were reported during the course of the study; one patient developed diarrhea (for 2 days from the beginning of therapy) that had no will to continue the treatment. Diarrhea improved after self-cessation of treatment. One patient complained about GI disturbances which resolved by curcumin administration after meal. No statistically significant changes in urea (p=0.73) and creatinine (P=0.13) were observed at baseline and 2 months after administration (Table 3).

**Table 3:** Comparison of laboratory parameter changes between placebo and treatment groups

	Placebo	Curcumin	p	95%CI
∆TC±SD	26±19.85	18.40±51.17	0.63^a^	-39.90-24.70
∆LDL-C±SD	21.25±19.88	34.15±45.01	0.36^a^	-16.11-41.92
∆HDL-C±SD	0.85±11.14	-2.06±5.98	0.38^a^	-2.92-3.29
∆VLDL±SD	1.62±8.51	5.84±7.16	0.27^a^	-3.64-12.08
∆TG±SD	3.57±44.04	25.76±31.30	0.14^a^	-8.31-52.70
∆FBG±SD, median (IQR)	-11.07±27.66, -5.00 (-25.50-7.50)	18.56±41.05, 3.50 (-12.50-40.50)	0.16^b^	-
∆hs-CRP±SD, median (IQR)	0.30±0.64, 0.05 (-0.07-0.63)	1.54±4.89, 0.06 (-0.16-0.50)	0.91^b^	-
∆Urea ±SD	2.95±9.73	1.03±11.76	0.64^a^	-10.37-6.53
∆Cr ±SD	0.02±0.08	-0.06±0.16	0.08^a^	-0.18-0.01

Values are mean ± SD or median (IQR). CI, Confidence Interval.

TC, total cholesterol; LDL-C, low-density lipoprotein cholesterol; HDL-C, high-density lipoprotein; VLDL, very low-density lipoprotein cholesterol; TG, triglycerides; FBG, fasting blood glucose; IQR, interquartile range; hs-CRP, high-sensitive C-reactive protein; Cr, creatinine.

a Two Independent- Samples t-tests,

b Mann-Whitney test.

## Discussion

In the current study, curcumin significantly reduced serum levels of LDL and triglycerides compared with baseline. But there was no significant difference in comparison to the placebo group. Levels of HDL-C increased and levels of total cholesterol decreased with curcumin but not to a significant degree. Nonsignificant differences between the study groups is probably due to small sample size. The within group statistical analyzes revealed significant differences in some lipid variables before and after curcumin treatment. The results can be due to comparing patients with themselves as a control in order to eliminate confounding factors. 

Curcumin was reported to have health benefits and potential cardioprotective effect (7, 11, 12). Cardioprotection can be attributed partly to lipid lowering and anti-inflammatory abilities. Taking into consideration the above results and reported effects in the literature curcumin can have a favorable effect on traditional and nontraditional cardiovascular risk factors. Quiles *et al.* reported cardioprotective effect of curcumin in atherosclerotic rabbits. They stated curcumin prevents lipoperoxidation of subcellular membranes (11). Another experimental study suggested that oral curcumin prevents LDL oxidation and has hypocholesterolemic effects in rabbits received diet in high cholesterol. They also claimed lower dose (1.6 mg/Kg) of curcumin has better control of lipid profile than higher dose (3.2 mg/Kg) (13). Another research study indicated the antiatherogenic effect of low-dose curcumin in apoE/LDL receptor double knockout mice. In this assessment, curcumin did not change cholesterol and triglyceride levels (14). Another investigator reported that curcumin significantly decreased serum TG, TC, and LDL-cholesterol in rats fed with a high fat diet for 8 weeks (15). 

Soni and Kuttan reported that daily consuming of 500 mg curcumin for 7 days significantly decreased serum cholesterol and increased HDL in 10 healthy volunteers (16). In another clinical study, 12 healthy subjects received a daily dose of 20 mg curcumin for 30 days. LDL decreased and HDL increased markedly in those healthy individuals (17). Baum *et al.* studied 31 subjects with Alzheimer disease in a six month double-blind, randomized control trial (RCT). The patients randomized to three groups: 1,000 mg or 4,000 mg daily doses of curcumin or placebo. They reported that curcumin did not significantly alter serum concentrations of cholesterol or triglyceride (18). Alwi *et al.* in a double-blind RCT examined the effect of curcumin on lipids in ACS patients. The results did not express statistical significant difference between the curcumin and the placebo group after 2 months of treatment (19). The recent study recruited 24 healthy humans receiving a total of 500 mg or total of 6000 mg curcumin in 7 days. Results showed cholesterol and triglyceride serum concentrations significantly decreased in a dose dependent manner (20). 

Detected changes of lipid profile (*e.g*. LDL & TG) in the current study are compatible with what Ramirez-Tortosa *et al*. and Pungcharoenkul and Thongnopnua reported. According to researches, suggested mechanisms of cholesterol reduction by curcumin include; inhibition of intestinal absorption, up-regulation of the LDL receptor and cholesterol 7a-hydroxylase, conversion of cholesterol into bile acids and fecal excretion of bile acids and cholesterol (21, 22). 

Controversial observations from studies conducted in different population and used different doses have been reported. The studies reporting hypolipidemic effects were not RCT and were done in healthy subjects. In addition, they were short trials using different doses of curcumin and not concurrently evaluating all components of the lipid profile. These differences between previous studies and this trial may explain the discrepancies in the reported results.

Curcumin is found to be associated with hypoglycemia in streptozotocin induced diabetic rats (22). Curcumin activated PPAR-γ and lowered blood glucose levels in diabetic mice (23, 24). It also enhanced insulin release by induction of *β*-cell electrical activity (25). Other investigators demonstrated that the dietary curcumin supplement improved insulin resistance and hyperglycemia in diabetic mice with a simultaneous increase in plasma leptin and insulin levels (26). In the present study blood glucose were decreased in the curcumin group and increased in the placebo group, although not statistically significant.

One of diverse mechanism by which curcumin mediates protection against CVDs is anti-inflammatory activities. Experimental study results of cardiopulmonary bypass and cardiac ischemia and reperfusion (I/R) suggested that curcumin considerably decreased proinflammatory cytokines and subsequent cardiomyocytic injury (27). CRP, as a nontraditional cardiovascular risk factor, is a well known predictor of CVDs. Nuclear factor-κB pathway Regulates CRP (28). Curcumin is a potent modulator of transcription factors such as NF-κB that exhibit critical role in signal transduction pathways in inflammatory illnesses. So curcumin has a favorable effect on chronic inflammatory disease; inflammatory bowel disease, rheumatoid arthritis and Alzheimer (8, 29). A pilot study reported curcumin clinically improved patients with inflammatory bowel disease in addition to decrease of sedimentation rate and CRP level (30). This clinical trial was not a double-blind placebo-controlled study. In a double-blind RCT, curcumin’s antirheumatic activity was compared to phenylbutazone. Curcumin with daily doses of 1200 mg for 2 weeks improved clinical symptoms in patients with rheumatoid arthritis (31). Another clinical trial evaluated the effect of curcumin as a maintenance therapy in 82 patients with quiescent ulcerative colitis. Subjects received daily doses of 2 g curcumin for six months and experienced improvement response was subjectively indicated by clinical activity index and objectively reported by the endoscopic index (32). other clinical studies (33-35) reported curcumin efficacy in clinical inflammatory processes but they did not measure inflammatory biomarkers. Results of current study verify these findings as curcumin therapy was associated with improvement in hs-CRP values. However, significant difference was not detected compared to baseline or the placebo group. Clinical studies, that addressed the curcumin’s anti-inflammatory properties taking into consideration of curcumin poor bioavailability (36), used daily doses of 1200 mg-2 g. Patients did not report any serious side effects. According to the results of previous studies, curcumin with doses up to 12 g is well tolerated (10). 

Regarding observed outcomes, we concluded that small sample size was a major limitation of this research, although it should be noted that this study was a pilot study. However, studies with larger number of patients may lead to decisive results. 

## Conclusions

According to the results, curcumin seems to be a potential candidate for decreasing cholesterol and triglyceride levels. Nonetheless, it has no appreciable effect on inflammatory biomarkers in patients with CAD. Further and larger human studies need to be done in order to establish a detailed assessment of metabolic effects and or anti-inflammatory actions of curcumin.
